# Applying Hickam’s dictum: a case of adult-onset LGMD2I muscular dystrophy and long QT syndrome

**DOI:** 10.1093/rap/rkad059

**Published:** 2023-07-10

**Authors:** Ali S F Sheikh, James B Lilleker, Hector Chinoy

**Affiliations:** Department of Medicine, Royal Oldham Hospital, Northern Care Alliance NHS Foundation Trust, Manchester, UK; Manchester Centre for Clinical Neurosciences, Northern Care Alliance NHS Foundation Trust, Manchester Academic Health Science Centre, Salford, UK; Division of Musculoskeletal and Dermatological Sciences, Faculty of Biology, Medicine and Health, The University of Manchester, Manchester, UK; Division of Musculoskeletal and Dermatological Sciences, Faculty of Biology, Medicine and Health, The University of Manchester, Manchester, UK; Department of Rheumatology, Salford Royal Hospital, Northern Care Alliance NHS Foundation Trust, Manchester Academic Health Science Centre, Salford, UK

Key message

- Genetic myopathies may mimic FM or myositis and, rarely, can manifest with dual inheritance.

Dear Editor, Muscle weakness and myalgia with associated hyperCKaemia is a common clinical presentation in both idiopathic inflammatory myopathy and other neuromuscular disorders. Likewise, cardiomyopathy can be a feature but may also be seen in muscular dystrophies. Although clinicians often rely on Occam’s razor when evaluating a case, it is not uncommon for a patient to have multiple diseases which together explain the presentation; so-called Hickam’s dictum. We describe the case of a young patient with muscle weakness, cardiomyopathy and hyperCKaemia, where a single unifying diagnosis was not forthcoming.

A 29-year-old health-care assistant with a 2-year history of gradually worsening myalgias, tiredness and exercise intolerance after her second pregnancy was reviewed in the specialized neuromuscular clinic at Salford Royal Hospital, UK. Over the preceding 6 months, she had started to crawl upstairs and needed to support her thighs with her arms when rising from a sitting position. She suffered several falls and severe cramps in her calves. She occasionally choked on saliva and suffered frequent snoring, unrefreshed sleep and orthopnoea. She continued to work but avoided strenuous tasks.

During her earlier years, she found it easier to walk on tiptoes, which she did for several years; however, this never posed a problem significant enough to be brought to medical attention. She experienced numerous falls during sports in school. At 10 years of age, during pre-operative assessment for grommet insertion in her ear, she was found to have long QT syndrome associated with a heterozygous mutation, c.1664G>A (p.Arg555His) in the *KCNQ1* gene. Subsequently, she was fitted with an implantable cardioverter defibrillator but remained asymptomatic. There was no family history of autoimmune or neuromuscular disorders.

Physical examination revealed short stature, subtle facial weakness, low-set ears, small jaw, tongue hypertrophy, fifth finger clinodactyly and calf hypertrophy (Fig. 1A and B). She was able to stand on tiptoes but not on heels and had proximal weakness in both upper and lower limbs and a weak ankle dorsiflexion. Gower’s sign was positive. There was no scapular winging.

**Figure 1.**
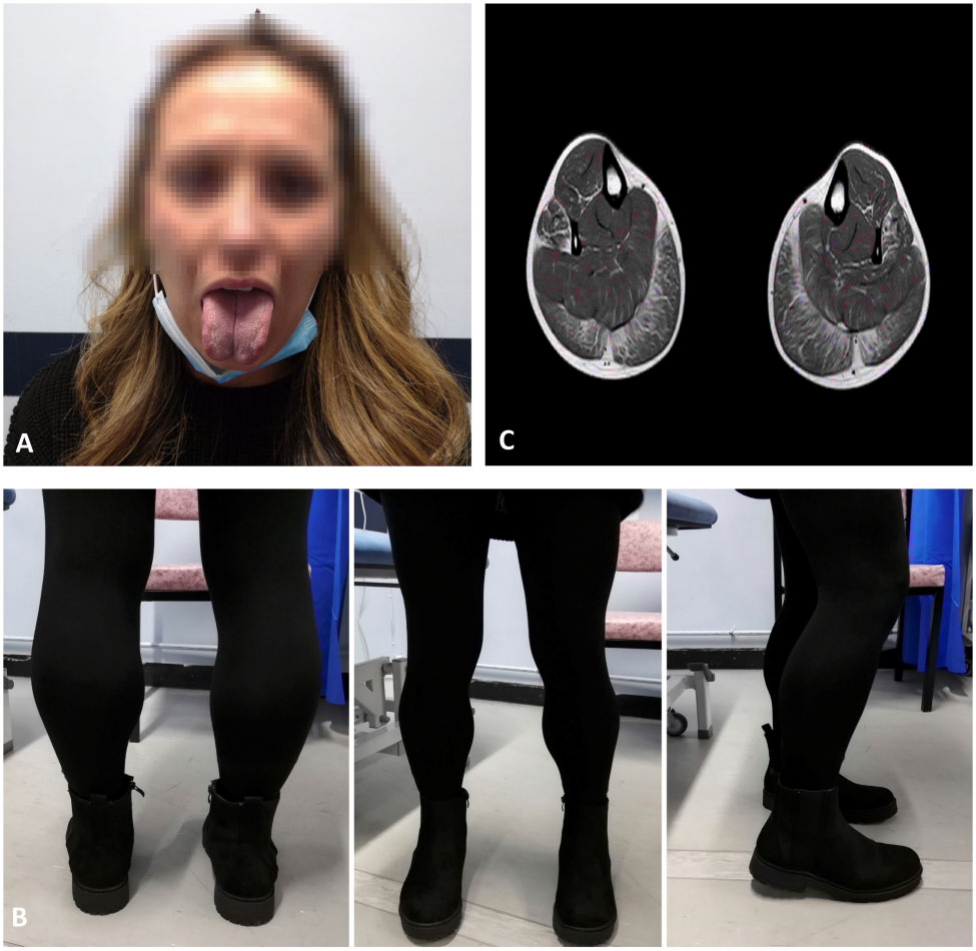
Key features of limb girdle muscular dystrophy type R9/2I (FKRP myopathy). (**A**) Tongue hypertrophy. (**B**) Calf hypertrophy. (**C**) Diffuse streaking of thigh musculature, with generalized fatty replacement

Investigations showed creatinine 37 µmol/l (normal range 45–84 µmol/l) and creatine kinase 1863 IU/l (normal range 25–200 IU/l). The extractable nuclear antigen panel, myositis-specific autoantibodies and α-glucosidase levels were normal. Muscle biopsy showed abnormal variation in fibre size, without any features of an inflammatory, dystrophic or myofibrillar myopathy. Special stains showed normal expressions of dystrophin, sacroglycan and spectrin.

Given the emerging features of a myopathy on the background of long QT syndrome, a unifying diagnosis of Andersen–Tawil syndrome was considered. However, no pathological variants of *KCNJ2/5* genes were detected. Further evaluation with MRI of the lower limbs (Fig. 1C) raised concern about limb girdle muscular dystrophy (LGMD), and additional genetic testing confirmed homozygosity for a pathogenic variant, c.826C>A p.(Leu276ile) in the *FKRP* gene, causative of LGMD type R9/2I (LGMDR9/2I). She found pregabalin beneficial for pain but needed an ankle–foot orthosis and was referred for genetic counselling, speech and language therapy and ventilatory assessment.

Differentiating LGMD from other inherited and acquired myopathies, including idiopathic inflammatory myopathy, can be challenging. In LGMD, muscle MRI shows prominent atrophy and fat replacement at presentation, often with selective involvement of certain muscles and striking sparing of other, often adjacent, muscles. In idiopathic inflammatory myopathy, fatty infiltration is usually a late-stage feature and tends to be less selective of the muscles. Muscle biopsy features can be misleading, because inflammation is frequently seen in patients with LGMD.

The prevalence of LGMD2I is unknown. The age of onset ranges from birth to 70 years. It follows an autosomal recessive inheritance [1]. Initial symptoms can include difficulty in walking, reduced performance at sports, myalgias and burning calves, mimicking FM and metabolic myopathies. In a recent review of similar patients in Mayo Clinic medical records, 50% of subjects had dual inherited myopathy, whereas 14% had coexistence of inherited myopathy with idiopathic inflammatory myopathy [2]. LGMD2I is associated with an increased risk of cardiomyopathy and respiratory insufficiency [3–5]. The history of implantable cardioverter defibrillator insertion in our patient with *KCNQ1* mutation caused confusion, because several channelopathies can manifest as long QT syndrome with or without non-cardiac abnormalities [6]. Her peculiar MRI findings narrowed the diagnosis.

Key features pointing towards LGMD included tongue and calf hypertrophy. Besides extrinsic chest wall insufficiency, upper airway disease can manifest as fatty tissue replaces the tongue musculature. This could result in obstructive sleep symptoms requiring specialist opinion. Given that the phenotype of LGMD can overlap with several muscle disorders, customized genetic testing is essential for accurate diagnosis. There is no definite treatment for LGMD, and an individualized care plan is needed based on disease manifestations. Physiotherapy and weight control measures can maintain mobility and prevent contractures. Tailored orthotic devices can help limitations. Genetic counselling of family members can aid timely diagnosis. A 6- to 12-monthly follow-up is reasonable to monitor muscle strength, functional status and disease progression.

Our case highlights the usefulness of Hickam’s dictum in clinical practice, particularly for complex cases, where separate genes can lead to cardiac and neuromuscular abnormalities. It accentuates the need to exclude mimics, such as inflammatory myopathies, with relevant antibody and biopsy testing, to ensure that inappropriate immunosuppression can be avoided.

## Data Availability

The data underlying this article are available in the article.

## References

[1] Kang PB , Feener CA, Estrella E et al LGMD2I in a North American population. BMC Musculoskelet Disord 2007;8:115. 18036232 10.1186/1471-2474-8-115 PMC2216011

[2] Granger A , Beecher G, Liewluck T et al Inherited myopathy plus: double-trouble from rare neuromuscular disorders. Neuromuscul Disord 2023;33:153–60. 36628841 10.1016/j.nmd.2022.12.009

[3] Ten Dam L , Frankhuizen WS, Linssen WHJP et al Autosomal recessive limb-girdle and Miyoshi muscular dystrophies in the Netherlands: the clinical and molecular spectrum of 244 patients. Clin Genet 2019;96:126–33. 30919934 10.1111/cge.13544

[4] Georganopoulou DG , Moisiadis VG, Malik FA et al A journey with LGMD: from protein abnormalities to patient impact. Protein J 2021;40:466–88. 34110586 10.1007/s10930-021-10006-9 PMC8190568

[5] Franekova V , Storjord HI, Leivseth G, Nilssen Ø. Protein homeostasis in LGMDR9 (LGMD2I) – the role of ubiquitin-proteasome and autophagy-lysosomal system. Neuropathol Appl Neurobiol 2021;47:519–31. 33338270 10.1111/nan.12684

[6] Nakano Y , Shimizu W. Genetics of long-QT syndrome. J Hum Genet 2016;61:51–5. 26108145 10.1038/jhg.2015.74

